# Quantitative Super-Resolution Microscopy Reveals the Relationship between CENP-A Stoichiometry and Centromere Physical Size

**DOI:** 10.3390/ijms242115871

**Published:** 2023-11-01

**Authors:** Yaqian Li, Jiabin Wang, Xuecheng Chen, Daniel M. Czajkowsky, Zhifeng Shao

**Affiliations:** 1 State Key Laboratory of Systems Medicine for Cancer, School of Biomedical Engineering, Shanghai Jiao Tong University, Shanghai 200240, China; lyq-551172@sjtu.edu.cn (Y.L.); zfshao@sjtu.edu.cn (Z.S.); 2 School of Chemistry and Chemical Engineering, Shanghai Jiao Tong University, Shanghai 200240, China; wjb0221@sjtu.edu.cn; 3 Shanghai Center for Systems Biomedicine, Shanghai Jiao Tong University, Shanghai 200240, China; chxch@sjtu.edu.cn

**Keywords:** centromere chromatin structure, CENP-A, stoichiometry, STORM, bio-macromolecules

## Abstract

Centromeric chromatin is thought to play a critical role in ensuring the faithful segregation of chromosomes during mitosis. However, our understanding of this role is presently limited by our poor understanding of the structure and composition of this unique chromatin. The nucleosomal variant, CENP-A, localizes to narrow regions within the centromere, where it plays a major role in centromeric function, effectively serving as a platform on which the kinetochore is assembled. Previous work found that, within a given cell, the number of microtubules within kinetochores is essentially unchanged between CENP-A-localized regions of different physical sizes. However, it is unknown if the amount of CENP-A is also unchanged between these regions of different sizes, which would reflect a strict structural correspondence between these two key characteristics of the centromere/kinetochore assembly. Here, we used super-resolution optical microscopy to image and quantify the amount of CENP-A and DNA within human centromere chromatin. We found that the amount of CENP-A within CENP-A domains of different physical sizes is indeed the same. Further, our measurements suggest that the ratio of CENP-A- to H3-containing nucleosomes within these domains is between 8:1 and 11:1. Thus, our results not only identify an unexpectedly strict relationship between CENP-A and microtubules stoichiometries but also that the CENP-A centromeric domain is almost exclusively composed of CENP-A nucleosomes.

## 1. Introduction

Centromeres play a crucial role in ensuring the integrity of chromosomal separation during mitosis as the genomic sites to which the kinetochore, and thereby the microtubules, attaches [1,2]. However, despite their fundamental importance, there is presently a very poor understanding of the structure and composition of centromeric chromatin [3,4,5]. It is known that the nucleosomal variant, CENP-A, localizes to a narrow region of the centromeric sequence, where it plays a dominating role in centromeric function, serving as the primary centromeric element that recruits the kinetochore proteins [6,7,8,9,10,11,12,13,14,15]. However, many fundamental details about CENP-A within centromeric chromatin remain incompletely known, including its density, degree of enrichment, and precise relationship with the assembled kinetochore [16,17,18,19]. In particular, with regards to the latter, early work observed that the number of microtubules in kinetochores of different physical sizes is essentially the same, with changes only to the spacing between the attached microtubules [19]. However, while it is known that the physical span of the CENP-A centromeric domain indeed matches the size of the kinetochore plate [19], it is not known whether the number of CENP-A-containing nucleosomes within this domain is also unchanged in centromeres of different physical sizes, which would reflect a strict relationship with the microtubule stoichiometry. Such information is not only important for understanding the structural relationship between the CENP-A nucleosomes and the kinetochore components, but also for understanding the structure of the CENP-A-enriched domain itself.

A recent study using conventional optical microscopy quantified the number of CENP-A molecules per centromere in human cells, finding that each centromere in mitotic chromosomes contains, on average, ~300 CENP-A proteins in U2OS cells [20]. However, owing to the limited spatial resolution, it was not possible to precisely delineate the size or structure of each CENP-A domain in this work. Further, this study estimated that the level of enrichment of CENP-A to H3 nucleosomes within centromeres is ~1:25 [20]. Yet this was based on the assumption that each centromeric region contains 1 Mb DNA [21] and not on actual measurements of the DNA content just within the CENP-A domains. Indeed, more recent work has shown that CENP-A generally binds within a limited span of the centromeric DNA, with some variability between different chromosomes [11,13,14]. Thus, the extent of enrichment of CENP-A-containing nucleosomes within centromeric chromatin is also presently not understood.

Here, we employ super-resolution optical microscopy (Stochastic Optical Reconstruction Microscopy, STORM) to determine the high-resolution ultrastructure [22,23,24,25,26] and stoichiometry [23,27,28,29,30] of CENP-A nanodomains, as well as DNA content, within human metaphase chromosomes. We find that each CENP-A locus is generally a single compact domain whose maximal lateral extent is, on average, 235 nm. In U2OS cells, we find that each domain contains, on average, 313 ± 10 CENP-A proteins, consistent with the earlier aforementioned work [20]. We find that there is some variability in the lateral size of the CENP-A domains of different centromeres (at most, 1.5-fold), yet the amount of CENP-A within these domains is essentially the same. Thus, CENP-A in physically smaller domains is more closely spaced, similar to the smaller microtubule spacing within smaller kinetochores [19]. In addition, by quantifying the DNA that overlaps the CENP-A domain, we estimate that the CENP-A:H3 ratio per domain is between 8:1 and 11:1. Thus, overall, these results show that the CENP-A domain is very highly enriched in CENP-A nucleosomes and, further, exhibits some variation in CENP-A density, the latter of which may ultimately lead to the variation in the inter-microtubule spacing within the corresponding kinetochores.

## 2. Results

### 2.1. Super-Resolution Imaging of Mitotic Chromosome Centromeric CENP-A Domains

**Figure 1:**
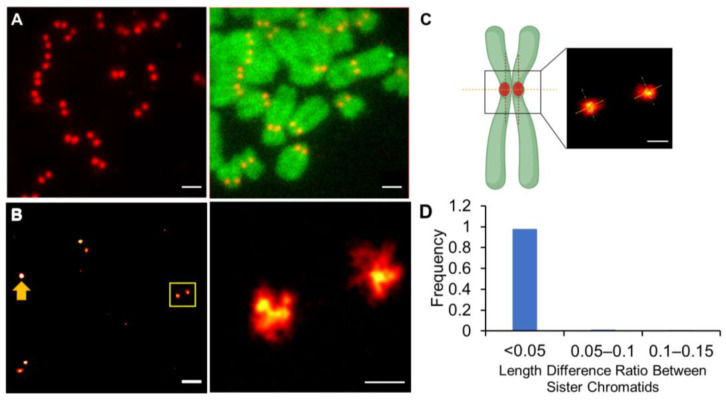
Structural analysis of the CENP-A domain in metaphase sister chromatids. (**A**) Conventional optical microscopy of Alexa-647-labeled CENP-A domains (red) reveals highly uniform labeling among the chromosomes from U2OS cells. The DNA is labeled with Atto 488 (green) attached to EdU incorporated into the chromosomes. Scale bar: 1 μm. (**B**) STORM images of the CENP-A domains in metaphase chromosomes within a single cell (**left**) and within a single pair of sister chromatids (**right**). The right panel is a magnified view of the boxed image in the left panel. The larger globular feature in the left region of the image on the left (arrow) is the TetraSpeck bead used to correct for drift during imaging. Scale bar: left, 1 μm; right, 200 nm. (**C**) Diagram showing the definitions of the axial and lateral lengths of the CENP-A domains. Scale bar: 200 nm. The figure was prepared using BioRender (URL: https://biorender.com (accessed on 20 June 2023). (**D**) Distribution of the differences in the ratios of the lateral lengths of CENP-A domains between sister chromatids as well as the ratios of the corresponding axial lengths of the domains between sister chromatids (*n* = 102).

Quantitative super-resolution microscopy has proven especially beneficial for studying the nanoscale organization of the cell nucleus [31,32,33,34]. As we discussed in a recent study, characterization of mitotic chromosome structure by localization-based super-resolution microscopy requires isolation of the native mitotic chromosomes from the cells since signals from fluorescence that is out of focus can reduce localization accuracy [26]. To maintain the structure and integrity of the mitotic chromosomal structure during isolation, we employed the cytospin method, which has been previously shown to effectively retain the mitotic chromosomal structure and composition [35,36,37]. In this method, metaphase-arrested cells are suspended in a hypotonic solution, then are centrifugated onto a coverslip with the sample maintained in a hydrated state throughout. This method has an additional advantage for our investigation as it ensures a similar co-planar orientation of the centromeres of different chromosomes on the coverslip, which aids our imaging-based quantitative comparison. Indeed, as shown in Figure 1A, imaging with conventional optical microscopy following immuno-labeling of CENP-A reveals two CENP-A-enriched domains (~200 nm in diameter) within each sister chromosome pair among the observed chromosomes, with a high degree of uniform labeling throughout the sample.

Much finer details of these CENP-A domains were obtained with STORM (Figure 1B and Figure S1, FRC resolution = 34 nm). The domains were found to generally entail a single compact domain, whose maximal (Feret) diameter was 235 ± 32 nm (ranging from 198 to 297 nm, Figure S2). Many of these domains exhibited smaller substructures; however, there was not a consistent number or organization of these substructures between different chromosomes. Overall, these domains were only slightly ellipsoidal (circularity = 0.72 ± 0.03, Figure S3), even when imaged with 3D STORM (Figure S4). We found that the lateral dimensions of the CENP-A domains of sister chromatids, as well as the axial dimensions of the domains of the sister chromatids, were highly similar; more than 98% of sister chromatids exhibited a CENP-A domain dissimilarity of less than 5% (Figure 1C,D). Thus, even at this high resolution, there is a high degree of structural identity between the CENP-A domains within sister centromeres.

### 2.2. Quantification of CENP-A Stoichiometry within Centromeric Chromatin

Localization-based super-resolution microscopy approaches can provide not only structural information but also measurements of stoichiometry [3,27,38,39,40,41]. The latter has typically been based on measuring the total number of detections associated with the biological complex and then dividing this by the number of detections for a single labeled component (for example, the number from a single secondary antibody for studies using primary and secondary antibodies, as in our study). However, the accuracy and precision of this calculation are significantly limited for samples that are highly labeled (which enables the highest possible resolution) owing to imprecise detection counts within a single diffraction-limited region [5,42]. To overcome this issue, we have recently developed an approach whereby the stoichiometry measurement is instead based on the number of detections (N_Feq_) detected during a quasi-equilibrium state of the system (between 400 and 600 s of imaging with Alexa 647 fluorophores) [43]. This method not only provides accurate measurements of stoichiometry, but also a 50-fold improvement in precision [44].

**Figure 2:**
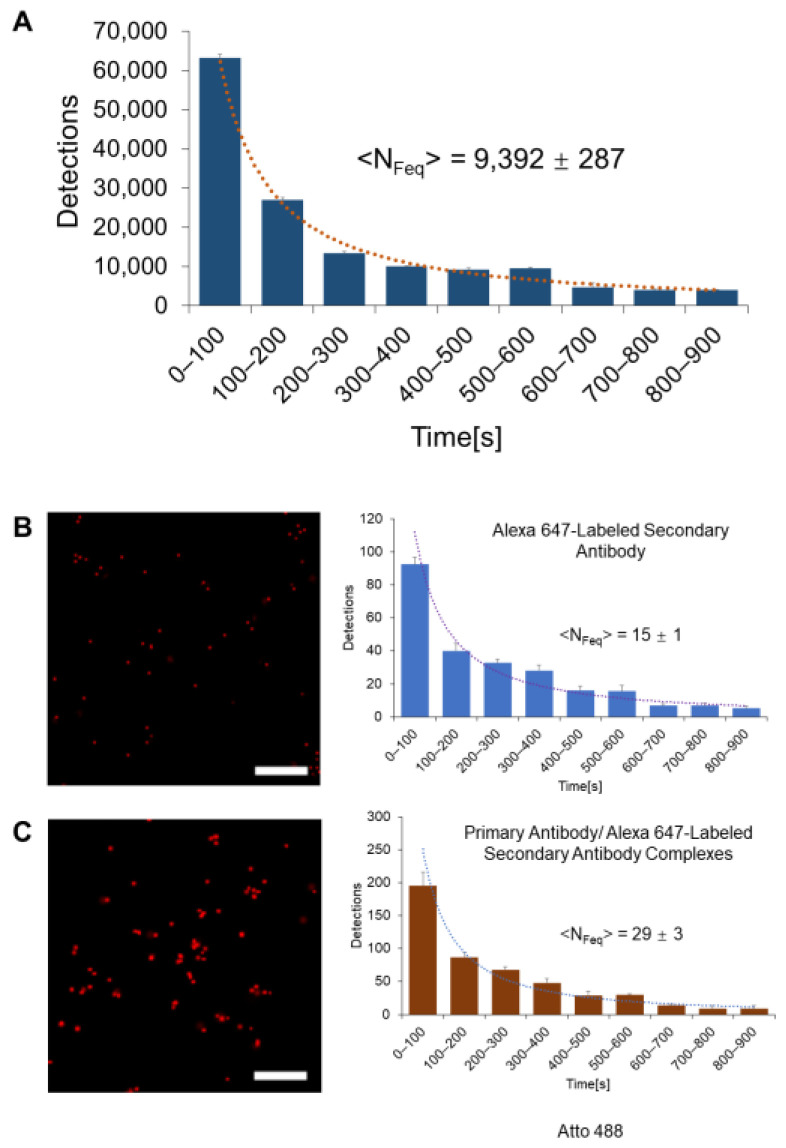
Quantification of the stoichiometry of the CENP-A domains. (**A**) Temporal evolution of detection measurements from a typical Alexa-647-labeled CENP-A domain. Shown is the average value of N_Feq_ measured from all CENP-A domains. (**B**) The detection number statistics for labeled antibodies and fluorescent molecules. For each case, a STORM image of a typical sample is shown on the left and the temporal evolution of the detections is presented on the right. Shown are data for the Alexa-647-labeled secondary antibody (**B**) and primary antibody/Alexa-647-labeled secondary antibody complexes (**C**) on a glass slide. The value of N_Feq_ shown in each figure is the average from all measurements. Scale bar: 400 nm.

**Figure 3:**
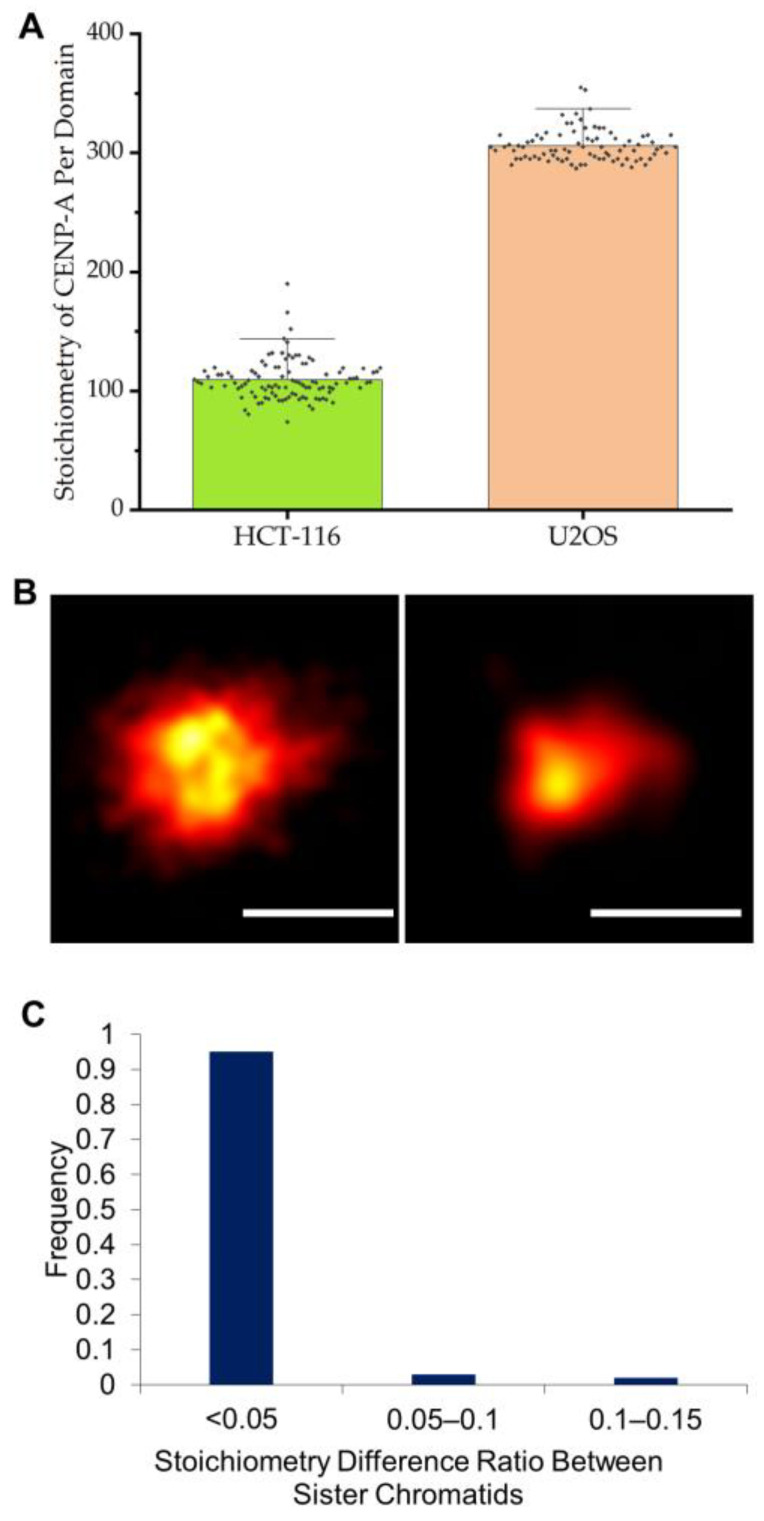
Stoichiometry of CENP-A within centromere chromatin. (**A**) Stoichiometry of each CENP-A domain in mitotic chromosomes from HCT-116 and U2OS cells. The dots in this figure represent the values from the individual measurements. (**B**) STORM images of typical CENP-A domains from U2OS (**left**) and HCT-116 (**right**) cells. Scale bar: 200 nm. (**C**) Comparison of the differences in CENP-A stoichiometry between sister chromatids in both cell lines.

As shown in Figure 2A for a typical domain, the temporal evolution of Alexa-647-labeled CENP-A within the centromeric domain indeed exhibits a quasi-equilibrium state between 400 and 600 s. Based on measurements during this interval, we determined that the mean number of accumulated detections (<N_Feq_>) is 9,392 ± 287 for the CENP-A domains. To translate these values into CENP-A stoichiometry, we made similar measurements of the number of detections within this time interval for samples of just the Alexa 647 secondary antibody as well as complexes between the primary and secondary antibodies (Figure 2B,C). Based on these measurements, we calculated that each U2OS centromeric CENP-A domain contains 313 ± 10 CENP-A molecules (*n* = 102) (Figure 3A, see Section 4), in excellent agreement with previous work [20]. This earlier work also noted that there is a roughly three-fold difference in the number of CENP-A in the centromeres of another cell line, HCT-116. We thus also quantified the centromeric CENP-A stoichiometry in HCT-116 cells and found that each domain contains 104 ± 12 CENP-A molecules (*n* = 96, Figure 3A,B), again in excellent agreement with this earlier work [20].

Finally, we examined the relative difference in CENP-A stoichiometry between each centromere of sister chromatids. We found that the vast majority of pairs differed by less than 5% (Figure 3C). Thus, both the overall size (Figure 1D) and stoichiometry (Figure 3C) of the CENP-A domains in opposing centromeres on sister chromatids are nearly identical in the vast majority of chromosomes.

### 2.3. Density of CENP-A Molecules in Centromere Chromatin

**Figure 4:**
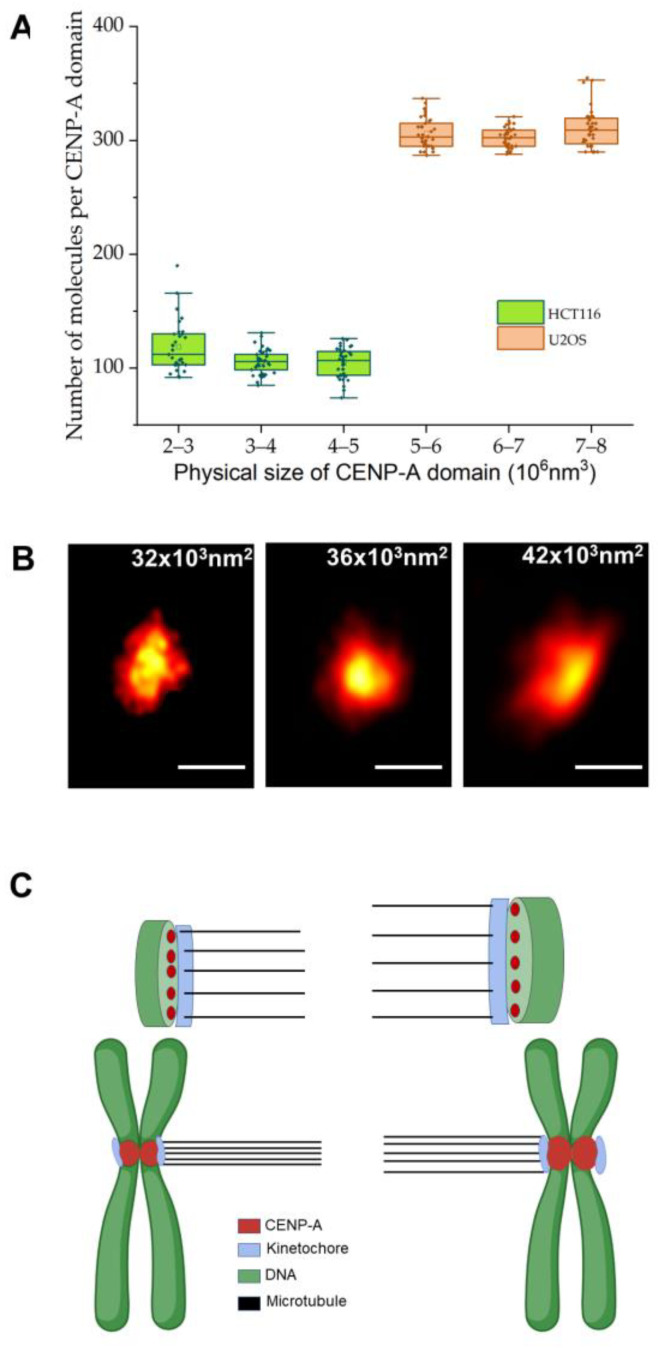
Density of CENP-A within centromeres. (**A**) The stoichiometry of CENP-A as a function of area in HCT-116 or U2OS cells. In both cell types, there is no significant difference in the CENP-A stoichiometry between domains that differ in area (*p* > 0.2, *t*-test). The dots in this figure represent the values from the individual measurements. (**B**) STORM images of CENP-A domains of different sizes within the U2OS cell line. Scale bar: 200 nm. (**C**) Schematic diagram showing the correspondence between the spacing between CENP-A nucleosomes and microtubules within the kinetochores suggested by our results. The figure was prepared using BioRender (URL: https://biorender.com (accessed on 20 June 2023).

Our ability to determine the precise size of the centromeric CENP-A domain and the stoichiometry of CENP-A within each domain provided an excellent opportunity to determine whether or not the stoichiometry changes with size. Strikingly, we found that, regardless of domain size, the number of CENP-A molecules within each domain was essentially unchanged (Figure 4A,B). This was true with both U2OS and HCT-116 cells (Figure 4A). Thus, smaller domains exhibit a higher density of CENP-A (Figure 4C).

**Figure 5:**
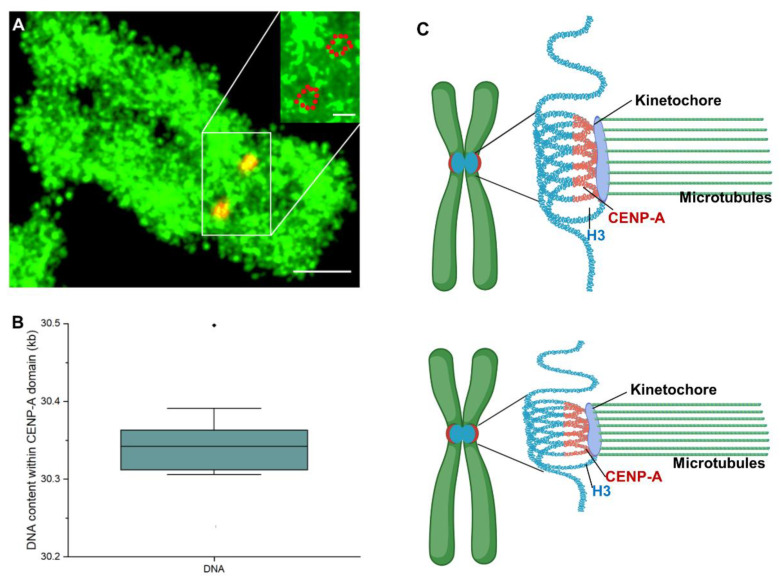
Quantification of the DNA content within CENP-A domains. (**A**) STORM image of the chromosomal DNA (green, Atto 488) and CENP-A (red, Alexa 647) domains. The overlap between the DNA and CENP-A is yellow/orange in this image. Inset (top right): zoomed-in view of the DNA image in which the locations of the CENP-A domains are delimited by red dots. Scale bar: main: 1 μm; inset: 200 nm. (**B**) DNA content that overlaps the CENP-A domains. (**C**) Structural model of a human chromosome centromere reflecting the high enrichment of CENP-A within the CENP-A domain. In this figure, the centromeric chromatin that contains H3 nucleosomes is in cyan. The figure was prepared using BioRender (URL: https://biorender.com (accessed on 20 June 2023).

We next sought to measure the DNA content within the centromeric CENP-A domain to enable an estimate of the extent of enrichment of CENP-A-containing nucleosomes. To this end, following a procedure that we developed previously for imaging chromatin DNA, we used EdU together with click chemistry to fully label chromosomal DNA [45]. Briefly, we trapped U2OS cells at the G1/S boundary using a double thymidine block and then added 10 μM of EdU throughout the entire S phase, which we (and others) have previously shown results in a highly uniform labeling of the whole genome [43,45,46] (Figure 1A and Figure S5), with an average incorporation of one EdU every 315 bp [45]. We performed click chemistry using the fluorophore Atto 488 to thereby label the DNA directly and enable measurement of the Alexa-647-labeled CENP-A domains within precisely the same samples (Figure 5A). The temporal evolution of the Atto-488-labeled DNA also exhibits a quasi-equilibrium state between 400 and 600 s (Figure S6) [40], from which we could measure the amount of Atto 488 and, thereby, the DNA content. We note that super-resolution images of the DNA in the metaphase chromosomes (Figure 5A) revealed an abundance of ~150 nm domains (157 ± 7 nm) throughout the arms of the chromosomes (Figure S7), consistent with our previous work [32] and with the work of others [47,48]. The preservation of these predominant 150 nm domains of higher-order structure within mitotic chromosomes from within the cell to these cytospin-prepared samples provides additional evidence for a minimal perturbation of the cytospin approach to the structure of mitotic chromosomes [35,36,37].

We found that the DNA content that overlaps the centromeric CENP-A domains was 30.3 ± 0.6 kb (Figure 5B). This value is in good agreement with recent studies, suggesting that these domains entail tens of kb within individual cells [11,12,13,14,15]. Assuming ~180 bp DNA per nucleosome (consistent with measurements from H3-containing nucleosomes [49]), we estimate that there are ~170 nucleosomes within this 30 kb DNA. Together with our measurement of CENP-A stoichiometry, we thus estimate that there are 156 CENP-A-containing nucleosomes (each containing two CENP-A molecules [20,43]) and 14 H3 nucleosomes. That is, the ratio of CENP-A nucleosomes to H3 nucleosomes in the CENP-A domain is 11:1. However, assuming ~171 bp DNA per nucleosome (consistent with measurements of CENP-A binding within the α-satellite region of human centromeres [15,50,51]), we estimate that there are ~160 CENP-A nucleosomes and ~20 H3 nucleosomes within this 30 kb DNA, yielding a ratio of 8:1 of CENP-A:H3 nucleosomes. Thus, these measurements suggest that the centromeric CENP-A domain is almost exclusively composed of CENP-A-containing nucleosomes.

## 3. Discussion

For the first time, we combined the high-resolution and quantitative capabilities of STORM to characterize the ultrastructure of the CENP-A domain within human centromeres and determine the stoichiometry and enrichment of CENP-A within this domain. Our measurements of stoichiometry are in excellent agreement with previous lower-resolution optical microscopy measurements, confirming the accuracy of our approach. Yet the additional high-resolution structural information of our results provides novel insight into both the structure and function of the CENP-A domain that was not possible to discern in previous work.

We found that, in U2OS cells, each CENP-A centromeric domain is essentially compact, is ~235 nm in average lateral extent, contains ~313 CENP-A molecules, and exhibits very little (less than 5%) variation in both size and stoichiometry between sister chromatids. We note that one concern with these measurements, particularly stoichiometry, is the issue of epitope accessibility, which is a common concern with immunofluorescence studies in general. However, the agreement between the values obtained here of the number of CENP-A proteins per domain in both U2OS and HCT116 cells and that obtained by Bodor et al. [20], who used three different methods to determine this stoichiometry, suggests that this effect may be minimally inhibiting in the present case.

Whether the high identity between sister chromatids that we observed here is owing to a high fidelity during the deposition process of CENP-A or a subsequent pruning mechanism that minimizes differences between the sisters is presently not clear. Nonetheless, we speculate that this exceptionally high identity is ultimately required to ensure balanced pulling forces imparted by microtubule-associated proteins during mitosis, thereby ensuring a proper separation of the sister chromatids [52,53,54]. However, we note that this idea requires that there is a very strict relationship between both the structure and stoichiometry of the CENP-A-containing domains and the assembled kinetochore structure, in particular in the number of microtubules bound per kinetochore, which ultimately determines these pulling forces [55]. Indeed, our work also provides evidence that supports such a strict relationship. Namely, we found that the number of CENP-A molecules per domain is essentially unchanged in domains of different sizes, similar to the invariance of microtubule number per kinetochores of different sizes observed previously [19]. A number of studies have shown that CENP-A interacts with the kinetochore through the CCAN complex [56,57], and other work has detailed the molecular mechanisms underlying the interaction between microtubules and the kinetochore [58,59], but the detailed structure of the kinetochore, especially the microtubule binding sites, presently remains unknown [60]. Our results suggest the intriguing possibility that the spacing of microtubule binding sites in the kinetochore is effectively mirrored, in some way, by the organization of CENP-A in the centromere chromatin. Our images reveal an essentially compact domain without any notable regularity in its organization that might correspond (eventually) to the microtubule binding sites in the kinetochore. The precise nature of this organization evidently requires even-higher-resolution interrogation than we obtained here (FRC resolution ~34 nm). Nonetheless, our results of the DNA content and CENP-A stoichiometry in these domains point to essentially exclusively CENP-A-containing nucleosomes within these regions. As depicted schematically in Figure 5C, this enrichment might not span a contiguous segment of the centromeric DNA, which was also evident in recent ChIP-Seq studies of CENP-A in human centromeres [11,61] as well as in previous fiber-FISH studies [15,62]. Such a structure must also allow for at least some change in local CENP-A density, as we found in both U2OS and HCT-116 cells. How this structure can exhibit flexibility in density yet also orderliness to ultimately effect microtubule spacing in the kinetochore remains a genuinely intriguing question for future work.

To conclude, our quantitative characterization of the CENP-A-containing domain within the human centromere provides basic physical parameters to inform the development of future structural models of the centromeric chromatin and kinetochore. In addition, our finding of an exceptionally high structural and stoichiometric identity between sister chromatids points to a high fidelity in the CENP-A deposition process or a subsequent pruning mechanism to minimize differences. Further characterization of these details will enable a better understanding of the molecular and physical mechanisms underlying the vital process of chromosomal separation during mitosis and how it may go wrong with pathological consequences.

## 4. Materials and Methods

### 4.1. Cell Culture, Synchronization, and EdU Incorporation

HCT-116 (ATCC, Manassas, VA, USA CCL247) and U2OS (ATCC, HTB96) cells were grown at 37 °C in an atmosphere of 5% CO_2_ in Dulbecco’s modified Eagle’s medium (Invitrogen, Carlsbad, CA, USA, C11995500CP) supplemented with 10% fetal bovine serum (BI, 04-002-1) and 1% penicillin/streptomycin (Invitrogen, 15140122). The cells were then digested with trypsin, collected by centrifugation at 1000 rpm, and resuspended in complete medium at a density of approximately 2 × 10^5^ cells per 10 cm culture dish.

To synchronize the cells, the cells were initially treated with 2 mM Thymidine for 18 h after passage to arrest the cells at G1/S phase. After release, the cells were cultured for 8 h in fresh medium and then treated again with 2 mM Thymidine for 18 h to enhance synchronization at G1/S phase [63]. Following release, the cells were incubated with 10 μM EdU (Sigma-Aldrich, St. Louis, MO, USA, T511293) for 8 h. Demecolcine (BBI, A606585) at a concentration of 0.1 μg/mL was added two hours after EdU treatment to arrest the cells in metaphase. Metaphase cells were collected by shake off, washed with PBS, and centrifuged at 1000× *g*/min for 3 min to remove residual demecolcine.

### 4.2. Preparation of Deposited Metaphase Chromosomes by the Cytospin Method

Glass coverslips (Cellvis, Mountain View, CA, USA, 051815) were first cleaned with detergent, rinsed with ultrapure water, and immersed in solutions of acetone, 1 M sodium hydroxide, and ethanol, each for 20 min, with rinsing in ultrapure water between each change of solution. Subsequently, the clean glass slides were placed in a 10% (*v*/*v*) poly-lysine solution (Sigma-Aldrich, P8920) for 30 min and then rinsed with ultrapure water.

The metaphase cells in PBS were then resuspended in 75 mM KCl, and the cells were subjected to this hypotonic treatment for 7 min at 37 °C. The cells were then incubated on ice for 1 min and added to the cleaned coverslip. The sample was then spun at 1500 rpm for 3 min using the Cytospin (Thermo Scientific, Waltham, MA, USA, A78300005). The sample with spread chromosomes was rmaintained in KCM buffer (120 mM KCl (Sigma Aldrich, P3911), 20 mM NaCl, 10 mM Tris HCl (Sigma Aldrich, T5941), 0.5 mM EDTA (Invitrogen, AM9620G), and 0.1% Triton X-100 (Sigma-Aldrich, T8787) for subsequent treatment.

We note that, although the chromosomes remained hydrated for the duration of this method, they nonetheless experienced some mechanical force during the centrifugation process that could have led to changes in the structure of the CENP-A domains. However, as the domains appeared to be compact, and the chromosomal arms were shown, following this treatment, to have retained the same ultrastructural features that are observed in cells [28], we believe that these mechanical perturbations induced only minor changes to the structure of the CENP-A domains in this work. This is further confirmed by images obtained by 3D STORM (Figure S4), which showed that these domains exhibit a roughly globular shape in all directions.

### 4.3. Preparation Samples for STORM

The metaphase chromosomes prepared with the cytospin method were fixed with 2% PFA (Adamas, Emeryville, CA, USA F8011) at room temperature for 10 min and then treated with 100 mM Glycine (Sigma Aldrich, G7126) for 10 min to terminate fixation, followed by incubation for 10 min in 0.5% (*v*/*v*) Triton X-100.

To perform the click reaction, the sample was incubated with the click reaction solution (2 mM CuSO4 (Sigma Aldrich, A600063-0500), 100 mM Sodium Ascorbate (Sigma Aldrich, 11140), 5 μM Azide Atto 488 (Thermo Fisher Scientific, A10436)), avoiding light, for 1 h in a humid box. After removing the reaction solution, the sample was washed with PBS three times. The samples were then incubated with freshly prepared 5% BSA (Equitech-bio INC., Kerrville, TX, USA, BAH68-1310) for 30 min.

To perform immunostaining of CENP-A, the samples were incubated overnight at 4 °C with the primary antibody, anti-CENP-A rabbit mAb (Thermo Fisher Scientific, H577.2), at a dilution of 1:100 in 5% BSA. The samples were then washed with PBST (PBS with 0.05% Triton X-100) and then incubated for 1 h at room temperature with the secondary antibody (diluted 1:200), Alexa Fluor 647 goat anti-rabbit IgG (Thermo Fisher Scientific, A27040,). The samples were then washed in PBST. Finally, to enable correction for drift during imaging, we incubated the sample with 100 nm TetraSpeck microspheres (Thermo Fisher Scientific, T7279) diluted 1:200,000 in PBS for 30 min.

### 4.4. STORM Imaging

The imaging buffers consisted of Buffer A (10 mM Tris (pH 8.0), 50 mM NaCl) and Buffer B (50 mM Tris (pH 8.0), 10 mM NaCl, 10% Glucose), GLOX solution (14 mg Glucose Oxidase (Macklin, Shanghai, China, G810483-250KU), 50 μL Catalase (Macklin, C6319, 17 mg/mL), 200 μL Buffer A), and 1 M MEA. We mixed 7 μL GLOX and 70 μL 1 M MEA with 620 μL Buffer B and vortexed it gently to mix on ice and then added this buffer to the sample. We used a commercial microscope system from Nikon Instruments (N-STORM) with a 50 mW 647 nm laser, a 50 mW 488 nm laser, a 100× objective lens (PlanApo TIRF, NA 1.49, Nikon, Tokyo, Japan), and an electron-multiplying charge-coupled device camera (iXon 3, Andor, Oxford Instruments, Abingdon, UK)). During imaging, we used a 20 ms exposure time and a 300 EM gain. We also used the perfect focus system (PFS) during the entire imaging period. The ThunderSTORM software (Version 1.3) was used to reconstruct the super-resolution images and quantify the detections [64]. Specifically, for the latter, we used the “detection” function in ThunderSTORM to measure the number of detections within the specific time ranges. For stoichiometry measurements, we determined the number of detections associated with each of the individual labeling molecules. In particular, we examined samples of just Alexa 647 secondary antibodies or Azide Atto 488; samples were prepared by diluting each stock solution 1:200, adding them to clean glass slides, and incubating them for 1 h, followed by washing and imaging (Figure 2B and Figure S6A). For complexes of the CENP-A primary antibody combined with Alexa 647 secondary antibody, a 1:100 dilution of the CENP-A primary antibody was first incubated on coverslips for 2 h, followed by incubation of the 1:200 diluted secondary antibody for 1 h, followed by washing and imaging (Figure 2C).

### 4.5. STORM Data Processing

Fluorophore positions were determined through the fitting of their point spread functions (PSFs) obtained from single-molecule emissions to a two-dimensional Gaussian intensity distribution using ThunderSTORM [64]. The resulting coordinates of the fluorescent labels were compiled and organized into localization lists. Subsequently, these localization lists were utilized to reconstruct the images employing ThunderSTORM, as performed previously [65,66]. ThunderSTORM was also used to count the number of localizations in the CENP-A domains from these images. To ensure spatial alignment, Tetraspeck microspheres (Invitrogen, F8801) were employed as reference markers during the STORM imaging.

### 4.6. Data Analysis

To quantify the reconstructed images of the CENP-A domains, the number of localized fluorescence molecules within each domain was first quantified. The number of detections per molecule within a 100 s window (equivalent to 5000 frames) was then calculated. The specific number of detections, N_Feq_, represents the sum of the single-molecule detections during the quasi-equilibrium period, namely from 400 to 600 s [40,43]. To convert N_Feq_ into stoichiometry, we performed a similar analysis of images of just the Alexa 647 secondary antibody, as well as complexes of the primary/Alexa 647 secondary antibody (Figure 2B,C). During this quasi-equilibrium imaging period between 400 and 600 s, one Alexa 647 fluorophore exhibits an average of 5 localizations [25,43], which agrees with our measurements of the secondary antibodies (which each contained, on average, 3 fluorophores [43,45]). The images of the primary/secondary antibody complexes indicate that there is an average of two secondary antibodies per primary antibody (Figure 2C), corresponding to the average value of N_Feq_ of 29 detections. The value obtained for each CENP-A domain divided by this value yields the amount of CENP-A per domain. A similar process was followed for the measurement of the DNA content that overlaps the CENP-A domain using the values obtained for Atto 488 (Figure S6).

Feret’s diameter is defined as the maximum distance between any two points along the boundary of a given shape and is also known as the maximum diameter of the shape. Circularity is defined as 4π × [Area]/[Perimeter]^2^, and it varies from 0 (indicating an infinitely elongated shape) to 1 (indicating a perfect circle). Both of these measurements were performed with ImageJ (Version 1.31).

## Data Availability

The data presented in this study are available on request from the corresponding author.

## Supplementary Materials

The following supporting information can be downloaded at: https://www.mdpi.com/article/10.3390/ijms242115871/s1.
