# Supplementary material for: Quantitative Super-Resolution Microscopy Reveals the Relationship between CENP-A Stoichiometry and Centromere Physical Size
Source: Int J Mol Sci. 2023 Nov 1;24(21):15871. doi: 10.3390/ijms242115871 (PMC10649757; doi:10.3390/ijms242115871)
Supplement: Supplementary file 1 [file ijms-24-15871-s001.zip › ijms-2613286-supplementary.pdf]

Supplementary Materials

Quantitative Super-Resolution Microscopy Reveals the Relationship between CENP-A Stoichiometry and Centromere Physical Size

Yaqian Li [1,†], Jiabin Wang [2,†], Xuecheng Chen [3], Daniel M. Czajkowsky [1,*] and Zhifeng Shao [1]

[1] State Key Laboratory of Systems Medicine for Cancer, School of Biomedical Engineering, Shanghai Jiao Tong University, Shanghai 200240, China
[2] School of Chemistry and Chemical Engineering, Shanghai Jiao Tong University, Shanghai 200240, China
[3] Shanghai Center for Systems Biomedicine, Shanghai Jiao Tong University, Shanghai 200240, China
* Correspondence: dczaj@sjtu.edu.cn; Tel.: +86-21-34206632
† These authors contributed equally to this work.

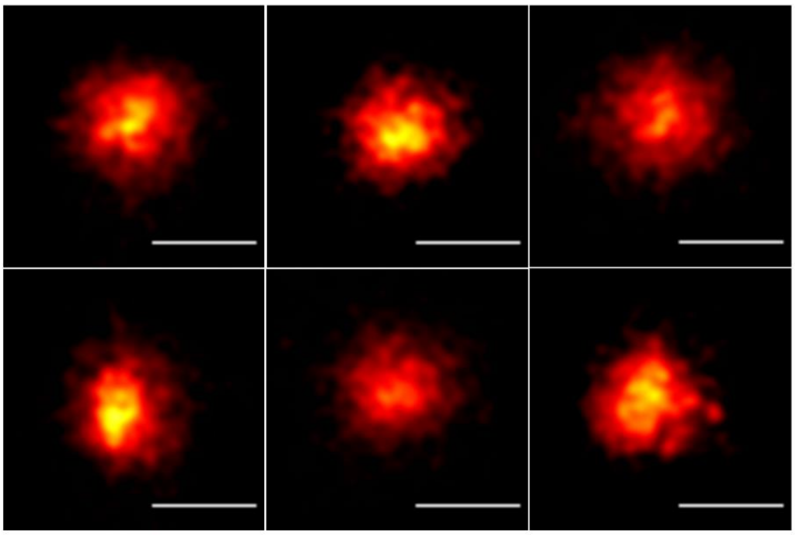

Figure S1. STORM images of individual CENP-A domains in U2OS metaphase chromosomes. Scale Bar: 200 nm.

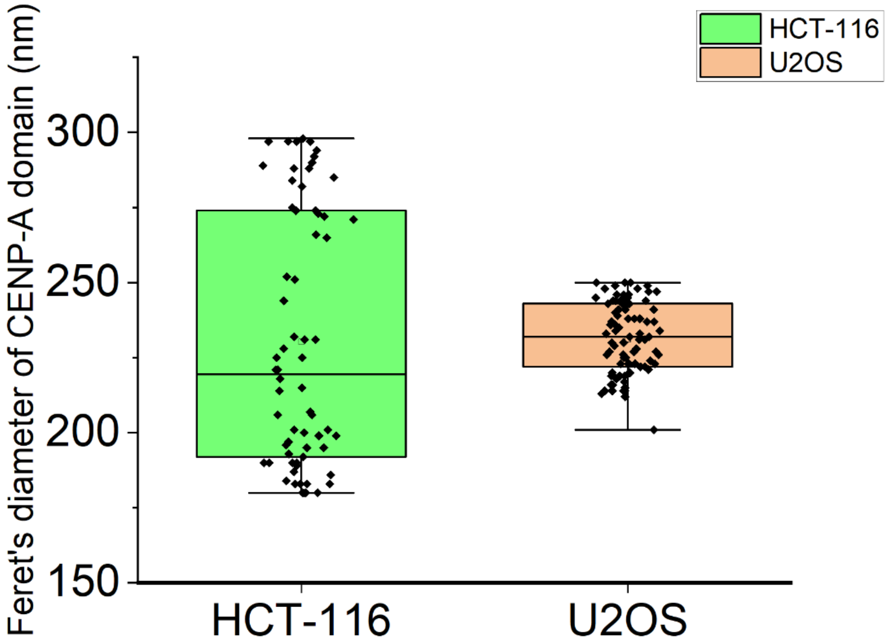

Figure S2. Feret's diameter of the CENP-A domain in HCT-116 and U2OS cells. The dots in this figure represent the values from the individual measurements.

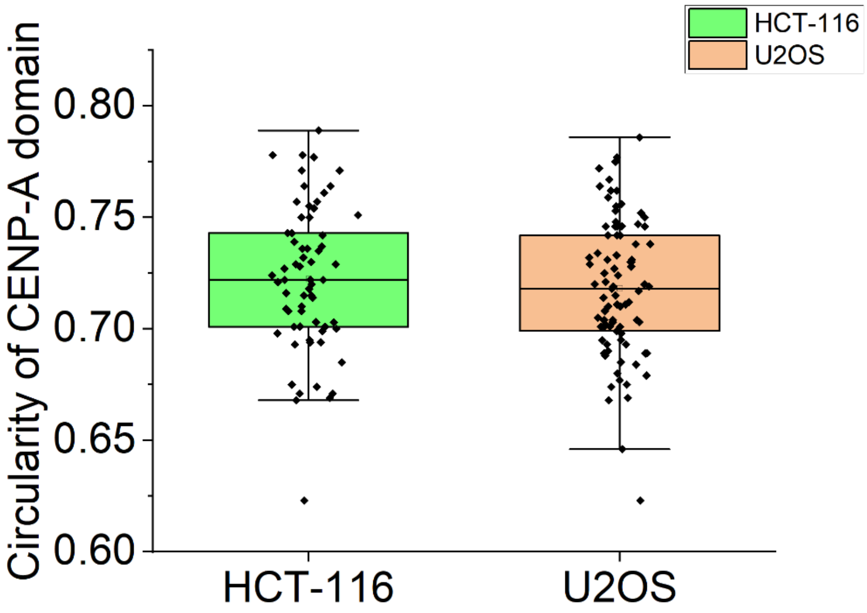

Figure S3. Circularity of the CENP-A domain in HCT-116 and U2OS cells. The dots in this figure represent the values from the individual measurements.

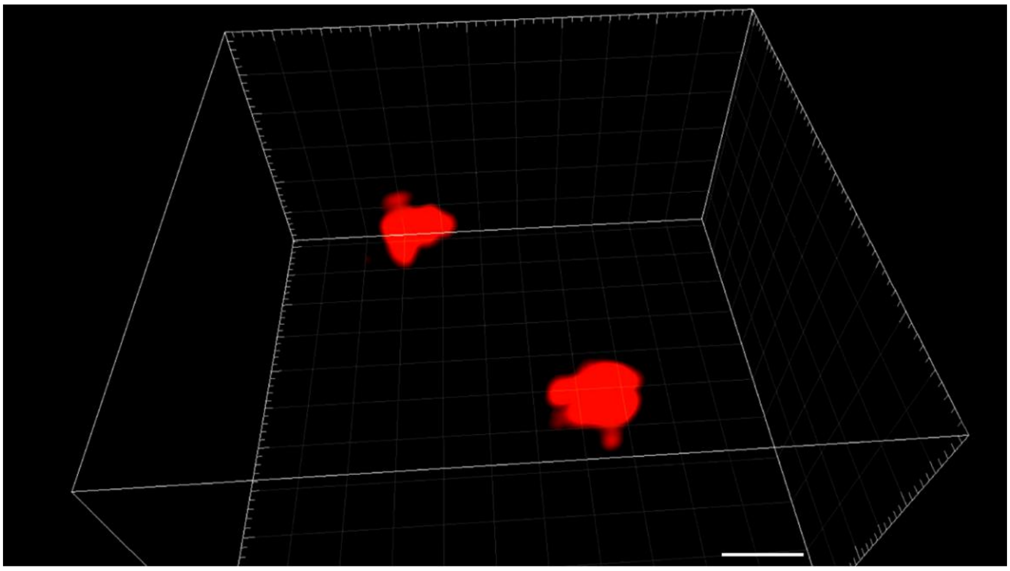

Figure S4. 3D STORM data of CENP-A domains on mitotic chromosomes. Overall, the size and shape of the domain is similar within the plane of the glass slide as perpendicular to the glass slide. Scale Bar: 200 nm.

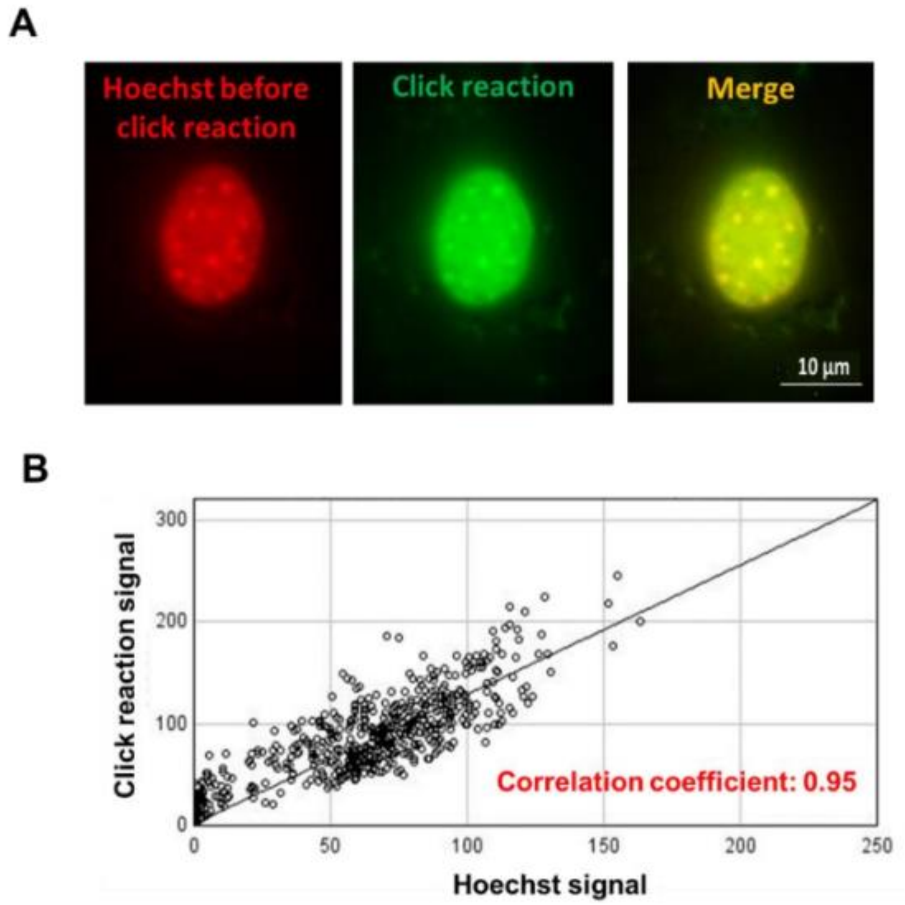

Figure S5. Evaluation of the uniformity of labeling of the genome by Alexa-647-EdU. (A) Hoeschst labeling (10 min, 5 µg/ml) of the genome gives a global view of the genome in the nucleus. After the click reaction there is, overall, a high overlap with the Hoeschst signal. (B) The scatter plot of the intensity profile reveals a high correlation between the conventional images of the nucleus before and after the click reaction.

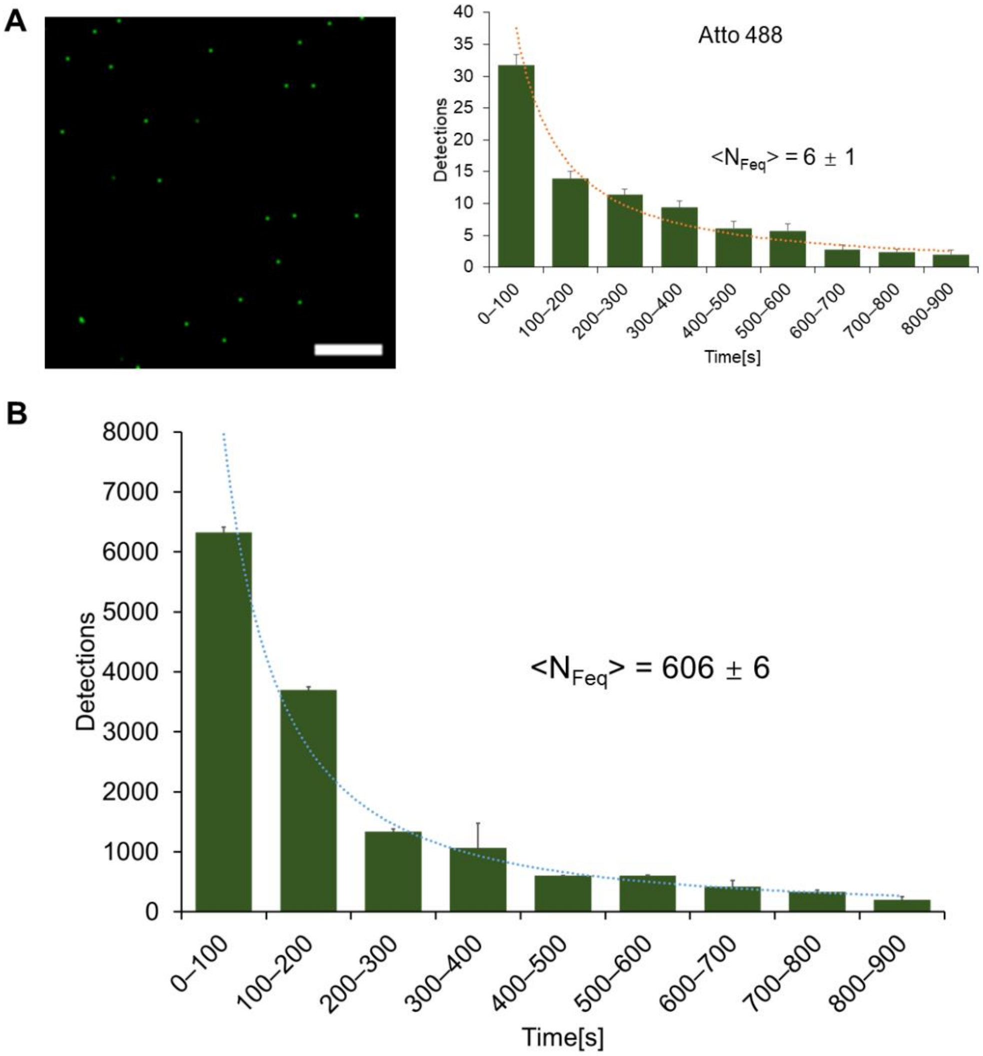

Figure S6. The detection number statistics for Atto 488 fluorophores and 488-labeled DNA**.** (**A**) STORM image of Atto 488 fluorophores is shown on the left and the temporal evolution of the detections is presented on the right. The value of N_{Feq} shown in the right is the average from all measurements. Scale bar: 400 nm. (B) An example of the temporal evolution of the number of detections of Atto 488-labeled DNA that overlaps a CENP-A domain. The value of N_{Feq} shown in the inset represents the average value from all measurements.

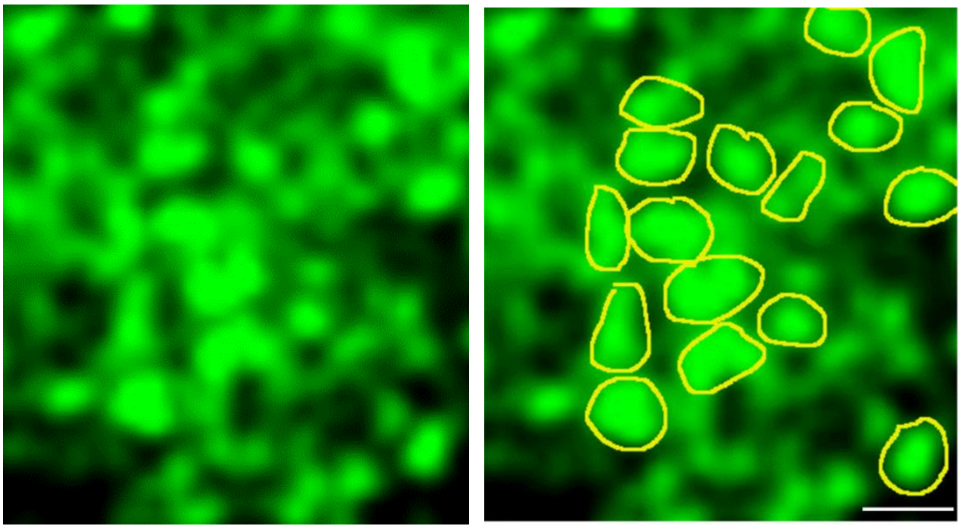

Figure S7. Identification of individual 150 nm domains in STORM images of the mitotic chromosomes. Shown are original images (left) and segmented images (right) that identify the 150 nm domains in images of U2OS mitotic chromosomes. Scale Bar: 200 nm
